# Revascularization after angiogenesis inhibition favors new sprouting over abandoned vessel reuse

**DOI:** 10.1007/s10456-019-09679-9

**Published:** 2019-09-04

**Authors:** Anthony Mukwaya, Pierfrancesco Mirabelli, Anton Lennikov, Muthukumar Thangavelu, Maria Ntzouni, Lasse Jensen, Beatrice Peebo, Neil Lagali

**Affiliations:** 1 Department of Ophthalmology, Institute for Clinical and Experimental Medicine, Faculty of Health Sciences, Linkoping University, 58183 Linköping, Sweden; 2 Mason Eye Institute, Ophthalmology-Retinal Vascular Service Hospital MA102C, Missouri, MO USA; 3 Department of BIN Convergence Technology & Dept PolymerNano Sci & Tech, Chonbuk National University, Jeonju, Republic of Korea; 4 Electron Microscopy and Histology Laboratory, Faculty of Medicine, Linköping University, Linköping, Sweden; 5 Division of Cardiovascular Medicine, Department of Medical and Health Sciences, Linköping University, Linköping, Sweden; 6 Department of Ophthalmology, Sørlandet Hospital Arendal, Arendal, Norway

**Keywords:** Neovascularization, Revascularization, Cornea, Regression, Empty basement membrane sleeves, Sprouting angiogenesis

## Abstract

Inhibiting pathologic angiogenesis can halt disease progression, but such inhibition may offer only a temporary benefit, followed by tissue revascularization after treatment stoppage. This revascularization, however, occurs by largely unknown phenotypic changes in pathologic vessels. To investigate the dynamics of vessel reconfiguration during revascularization, we developed a model of reversible murine corneal angiogenesis permitting longitudinal examination of the same vasculature. Following 30 days of angiogenesis inhibition, two types of vascular structure were evident: partially regressed persistent vessels that were degenerate and barely functional, and fully regressed, non-functional empty basement membrane sleeves (ebms). While persistent vessels maintained a limited flow and retained collagen IV+ basement membrane, CD31+ endothelial cells (EC), and α-SMA+ pericytes, ebms were acellular and expressed only collagen IV. Upon terminating angiogenesis inhibition, transmission electron microscopy and live imaging revealed that revascularization ensued by a rapid reversal of EC degeneracy in persistent vessels, facilitating their phenotypic normalization, vasodilation, increased flow, and subsequent new angiogenic sprouting. Conversely, ebms were irreversibly sealed from the circulation by excess collagen IV deposition that inhibited EC migration and prevented their reuse. Fully and partially regressed vessels therefore have opposing roles during revascularization, where fully regressed vessels inhibit new sprouting while partially regressed persistent vessels rapidly reactivate and serve as the source of continued pathologic angiogenesis.

**Electronic supplementary material:**

The online version of this article (10.1007/s10456-019-09679-9) contains supplementary material, which is available to authorized users.

## Introduction

Pathological angiogenesis can lead to serious progression of disease and is typically treated using molecular inhibitors of angiogenesis. Clinically, this strategy is widely used to limit retinal tissue damage in neovascular age-related macular degeneration (nAMD) and diabetic retinopathy [1–3], to avoid immune rejection in high-risk corneal transplantation [4, 5] or to prevent tumor growth and metastasis in various cancers [6–9]. Although anti-angiogenic therapy (to date principally focused on VEGF inhibition) can partially regress vessels to maintain useful vision [1–3] or achieve a modest survival benefit in cancer patients [10, 11], discontinuation of anti-angiogenic therapy or a gradual decline in therapeutic effect eventually ensues [3, 12]. This carries the risk of rebound, whereby angiogenesis is re-activated by the underlying inflammatory, hypoxic, or angiogenic stimulus [3, 13–20]. This angiogenic rebound or ‘revascularization’ of the tissue necessitates repeated or continued anti-angiogenic treatment; however, continued treatment comes at a cost, including adverse effects/toxicity of prolonged therapy [11, 21, 22], acquired drug resistance [23, 24], and an escalating economic and health care delivery burden of repeated treatments [25].

Mechanisms of tissue revascularization after initial angiogenic regression therefore deserve closer investigation, although to date relatively little is known about the manner by which vessels respond to a change in the tissue microenvironment from anti- to pro-angiogenic to mount a revascularization response. Earlier studies have examined revascularization of skeletal muscle [26, 27], the retina [28, 29], and the heart [30] following experimental injury; however, such vasculatures were not pathologically angiogenic in nature. In the eye, choroidal neovascularization in AMD and retinal neovascularization in proliferative diabetic retinopathy are caused by angiogenesis; however, even advanced clinical imaging methods have insufficient resolution to examine revascularization dynamics at the single-vessel level [3, 12]. Tumor vessels are similarly angiogenic, and although pre-clinical and clinical studies report tumor revascularization following stoppage of anti-VEGF treatment [9, 19, 20, 31], the detailed mechanisms by which revascularization may occur are less well studied. In a prior study by Mancuso and colleagues, revascularization of experimental tumors after a short duration (7 days) of anti-VEGF therapy was reported to occur by reuse of the empty basement membrane sleeve (ebms) remnants of regressed vessels [32]. Anti-VEGF therapy, however, is often administered over a prolonged period, for example in tumors [19, 20] or to treat nAMD [1]. It is unknown how a longer course of anti-angiogenic treatment affects the newly formed vessels, or how revascularization proceeds once the treatment effect subsides. In nAMD, a longer period between anti-angiogenic treatments [33] is becoming more common, driven primarily by economic considerations.

A major limitation of current models of revascularization is an inability to examine tissues in vivo. Analysis of different tissues post-harvest and processing precludes investigation of the dynamics of revascularization of the same vasculature. Typical dense vascular invasion within tumors also makes difficult an unambiguous discrimination between persistent and revascularizing vessels. Revascularization may also be influenced by factors such as the type and efficacy of anti-VEGF therapy, degree of inflammation present, and alternative pro-angiogenic factors that may be activated upon VEGF inhibition [34–41]. To address these multiple issues, we opted to investigate revascularization in the cornea, one of the first tissues used to study angiogenesis [42–45] and where anti-VEGF drugs originally developed for tumor treatment are used clinically [4, 5]. Advantages of the cornea as a model include its transparency and accessibility for in vivo examination, permitting longitudinal in vivo analysis of the same vessels over time [46]. Cornea models further allow a controlled and reproducible pattern and timing of angiogenesis [47–49], while the relatively thin corneal tissue permits characterization of the entire neovascularized region in vivo using high-resolution imaging methods. Here, we induced inflammatory angiogenesis in the rat cornea in a reversible manner [47] by suture placement to stimulate inflammation and angiogenesis, followed by suture removal to abruptly inhibit angiogenesis and induce vessel regression. This method of inhibiting angiogenesis is not solely dependent on interfering with VEGF signaling, as a multiplicity of endogenous inflammatory and angiogenesis inhibitors present in the cornea are activated upon suture removal, in attempt to restore the cornea’s normal avascularity [47, 48, 50]. Following a 30-day regression phase, angiogenesis inhibition was reversed by re-suturing the same cornea to induce a revascularization response, simulating discontinuation of an anti-angiogenic treatment in the presence of an underlying pathology.

Using this model, opposing roles of different vascular structures in the regressed vascular bed were observed, which were additionally sensitive to the duration of angiogenesis inhibition. The study elucidates for the first time the relative importance of different vessel phenotypes present after angiogenesis inhibition, and the phenotypic changes by which revascularization occurs. The results suggest that revascularization is principally mediated by rapid recovery of persistent, partially regressed vessels, and not by reuse of the fully regressed structures (the empty basement membrane sleeves) as conduits by the EC.

## Results

### A model for investigating revascularization dynamics in vivo

**Fig. 1:**
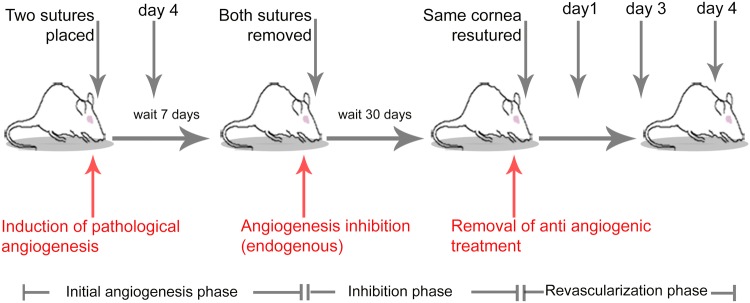
Rat cornea model of revascularization. An experimental model for reversibly inducing regression of angiogenic vessels in the cornea. The text in red color corresponds to the clinical interpretation of the experimental interventions. The phases in the model are separated into initial angiogenesis, angiogenesis inhibition, and revascularization. The gray-colored arrows indicate time points of longitudinal in vivo examinations in the same animal

To recapitulate phenomena of adult pathological angiogenesis and revascularization, a suture model of inflammatory corneal angiogenesis followed by vascular regression was used as a basis [47, 48]. Surgical nylon sutures placed into the rat cornea induced vessel sprouting from the preexisting limbal arcade toward the sutures during a 7-day period (initial angiogenesis phase). On the 7th day, sutures were removed to allow angiogenic vessels to regress naturally for 30 days (angiogenesis inhibition phase). After 30 days of regression, the same cornea was re-sutured to model discontinuation of angiogenesis inhibition (triggering a revascularization phase). Corneas were thereafter examined longitudinally up to 4 days into the revascularization phase (Fig. 1).

### Two types of vascular structures are present following sustained regression

**Fig. 2:**
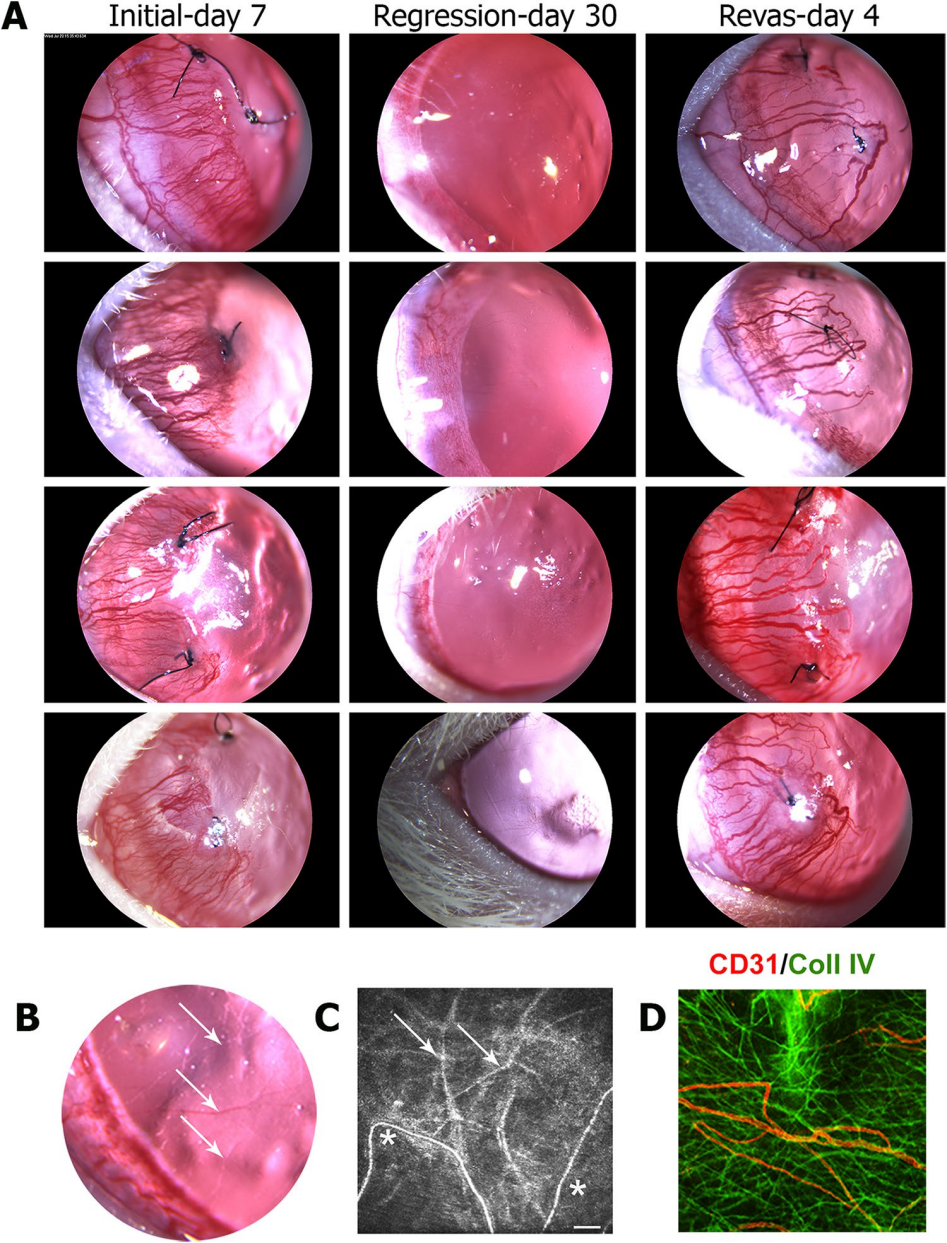
Two types of vascular structures are present following a period of substantial vascular regression. Each row represents longitudinal slit lamp images of the same rat cornea during initial angiogenesis (7 days following initial suture placement), after 30 days of regression (30 days after suture removal), and after 4 days of revascularization (4 days after suture re-placement). Vessels are hyper-dilated during revascularization but are less densely distributed than during initial angiogenesis at 7 days. Note an almost complete regression 30 days following suture removal. **b** Magnified region of a regressed vascular bed with a few persistent vessels indicated by arrows, identified by the presence of (severely restricted) blood flow. **c** IVCM image after 30 days of regression, indicating thin persistent vessels with flow (asterisks) and abandoned ebms without flow (arrows). **d** Immunofluorescence of a whole-mounted cornea after 30 days of regression, stained for vascular endothelium (CD31, red) and basement membrane marked by type IV collagen (Coll IV, green). Persistent vessels with flow expressed CD31 and Coll IV while ebms expressed only Coll IV

Suture placement stimulated inflammation and angiogenesis, with blood vessels extending to the sutures by day 7 of initial angiogenesis. Suture removal led to vessel regression, which was potent and substantial, with the cornea regaining transparency after 30 days of regression (Fig. 2a). Following regression, re-suture of the same cornea led to rapid revascularization, with the revascularized vessels observed as larger, hyper-perfused structures by day 4 of revascularization leading to a more aggressive vascular phenotype (Fig. 2a). A closer examination of the vascular bed after 30 days of regression indicated two distinct vascular structures within the tissue: partially regressed but persistent vessels barely visible in the slit lamp, which were severely constricted with markedly reduced flow (Fig. 2b), and fully regressed vessels with only empty basement membrane sleeves (ebms) as remnants that were invisible in the slit lamp but were clearly visualized by in vivo confocal microscopy (IVCM) as diffusely reflective, non-perfused vessel remnants (Fig. 2c). By immunohistochemistry, the ebms expressed type IV collagen (Coll IV) but not CD31, whereas the partially regressed persistent vessels with flow expressed both CD31 and Coll IV (Fig. 2d).

### Ebms pervade the tissue, do not express CD31, and do not support blood flow during revascularization

**Fig. 3:**
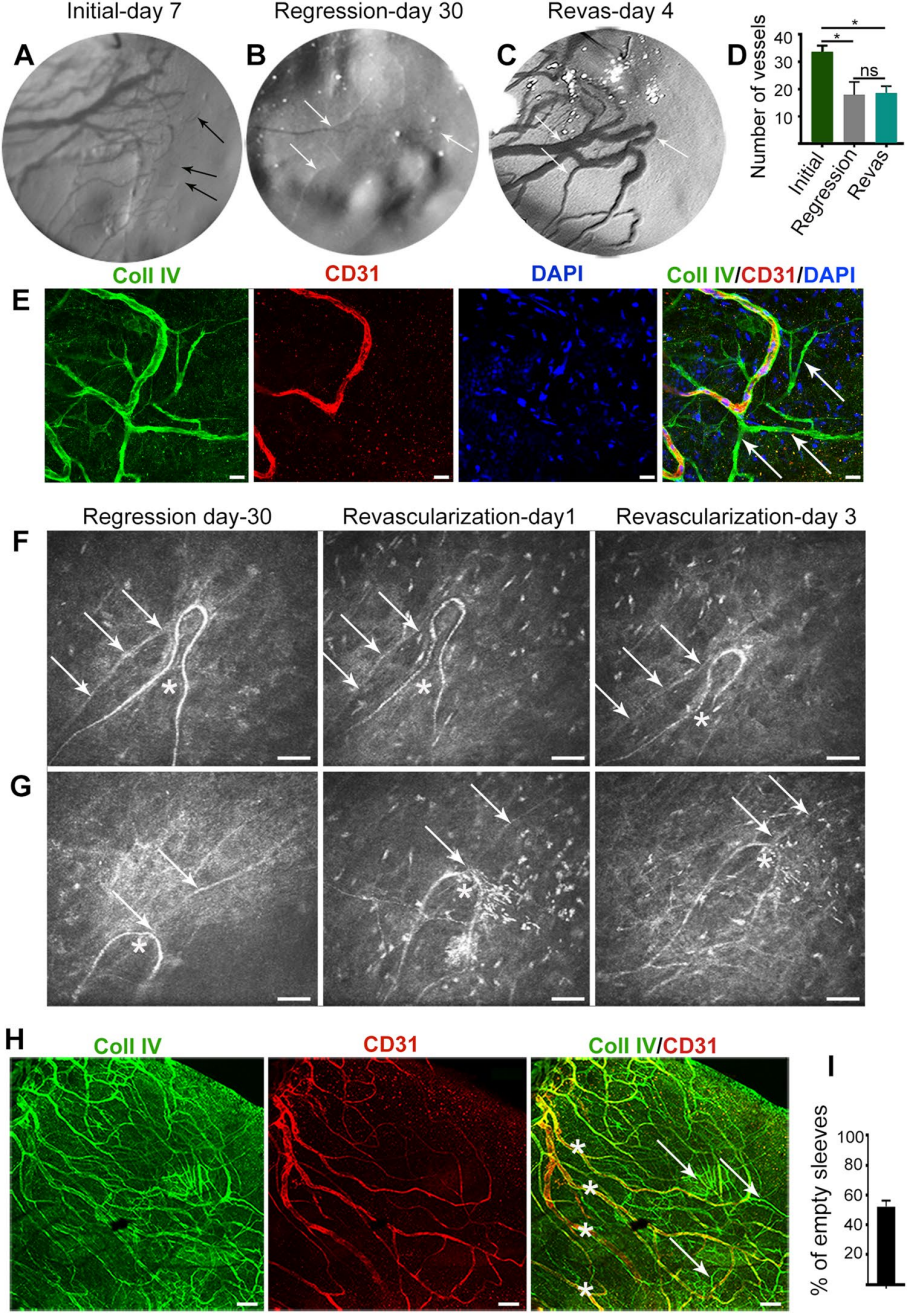
Persistent vessels support flow but ebms remain non-perfused during revascularization. **a**–**c** Serial slit lamp photos of the same cornea. On day 7 of initial angiogenesis, sprouting was apparent at the front of invading vessels with many thin, perfused sprout tips visible (**a** arrows). After 30 days of regression, only a few persistent vessels were visible (**b** arrows). (**c**) The persistent vessels hyper-dilated during revascularization, with arrows corresponding to the same persistent vessels observed in **b**. Note the absence of thin sprout tips at the leading edge of the persistent vessels in **c**. **d** The number of vessels per cornea quantified from slit lamp images was reduced in revascularization relative to initial angiogenesis, with (ANOVA *P* < 0.05), but no difference in vessel count was observed between day 30 and day 4 of revascularization. **e** Immunostaining at day 4 of revascularization revealed non-perfused sprout tips expressing Coll IV but not CD31, indicating these were ebms, while perfused vascular loops expressed Coll IV and CD31. **f** and **g** Longitudinal IVCM images of the same persistent vessels (asterisks) and ebms (arrows). After 30 days of regression, ebms could be differentiated from persistent vessels maintaining flow. After 1 and 3 days of revascularization, persistent vessels maintained flow, while ebms did not re-perfuse and remained dormant. Note the increase of infiltrating inflammatory cells starting on day 1. **h** Immunostaining of the revascularized cornea on day 4 revealed a network of ebms in the tissue (arrows) expressing Col IV but not CD31, while persistent vessels (asterisks) expressed both markers. **i** Quantification of CoIl IV+ CD31− structures versus all ColI IV+ structures in the revascularized cornea indicated ebms expressing only Coll IV comprised 52% of stained structures. The error bars in **i** represent SEM. Scale bars: **e** 20 µm, **f** and **g** 50 µm, **h** 100 µm. *n* = 5 and 3 for **d** and **i**, respectively

Comparison of longitudinal slit lamp images in the same cornea revealed that during revascularization, persistent vessels barely visible after 30 days of regression became hyper-dilated and re-perfused (Fig. 3b, c). The initially dense angiogenic vasculature that included many blood-filled sprout tips at the leading edge of vascular invasion (Fig. 3a, Supplementary Fig. 1) was not recapitulated during revascularization. No sprouts were observed at the leading edge of vessel invasion (Fig. 3c) and an overall reduction in the number of corneal vessels was apparent after regression and revascularization, relative to initial angiogenesis (*P* < 0.05 for both, Fig. 3d). The one-to-one correspondence of persistent and re-perfused vessels (Fig. 3b, c) resulted in a maintained number of vessels after regression and upon revascularization (*P* > 0.05, Fig. 3d). Immunostaining of whole-mounted corneas at day 4 of revascularization similarly indicated the absence of leading-edge perfused sprouts from persistent vessels (Fig. 3e). Persistent vessels expressed both CD31 and Coll IV, while sprouts from the initial phase of angiogenesis had fully regressed and only expressed Coll IV. Detailed immunohistochemical analysis revealed that CD31 expression of persistent vessels abruptly stopped at junctional points with ebms but did not extend onto the ebms (Fig. 3e, Supplementary Fig. 2A, B). Ebms were observed throughout the revascularized region with morphology varying from thin string-like structures to larger tube-like structures, in contact with wider persistent vessels (Fig. 3e, h, Supplementary Fig. 2A–C). Observed by live imaging (IVCM), the persistent vascular loops had strong flow, whereas ebms were without flow (Fig. 3f, g, Supplementary Fig. 2D, E, Supplementary Video 1). Longitudinal still images of the same vessels by IVCM (Fig. 3f, g) revealed that ebms although in physical contact with persistent vessels did not regain vascular endothelium (flow) or exhibit altered morphology during the revascularization phase. An invasion of inflammatory cells into the tissue was also visible by IVCM at day 1 of revascularization and onwards. Whole-mount immunostaining and co-localization analysis revealed that ebms expressing Coll IV but not CD31 comprised about half (52%) of the total basement membrane positive structures in the vascular bed, with persistent vessels co-expressing Coll IV and CD31 comprising the remainder (Fig. 3h, i).

### Persistent vessels undergo de novo angiogenic sprouting during revascularization, independent of ebms

**Fig. 4:**
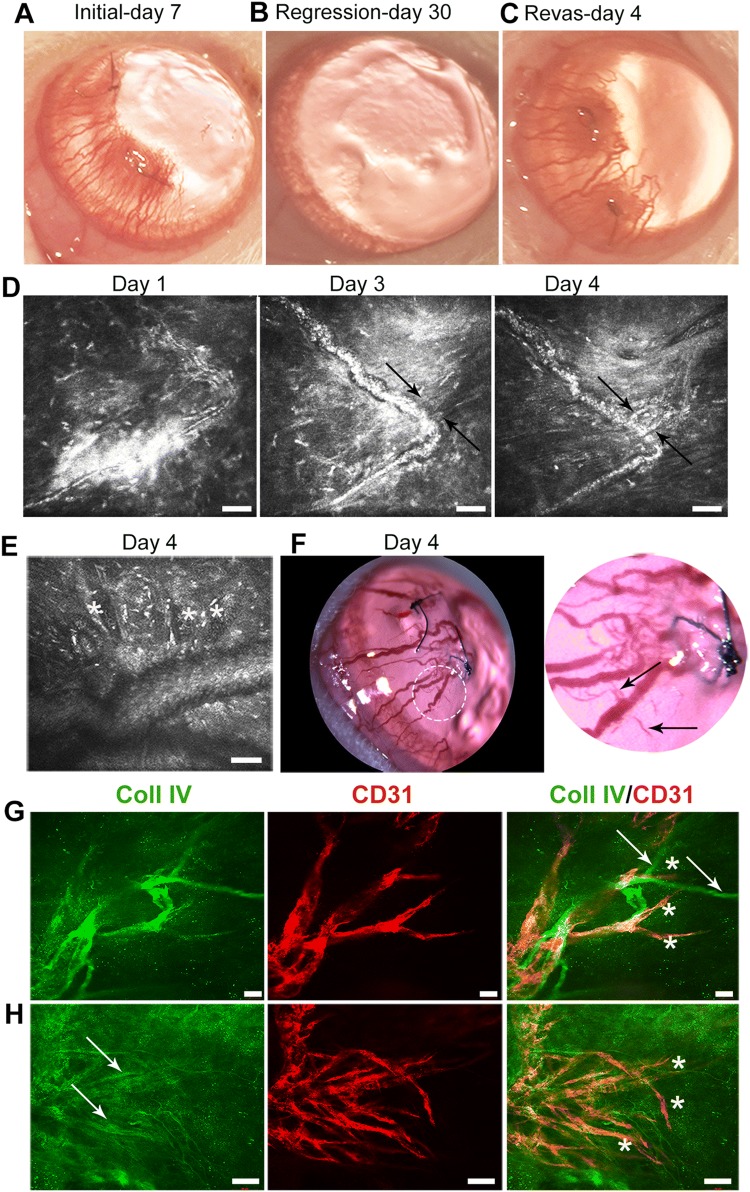
Sprouting occurs *de novo* from persistent vessels. **a**–**c** Longitudinal slit lamp examination of the same eye. **b** Vessels are barely visible after regression. **c** Persistent vessels hyper-dilate during revascularization. **d** Longitudinal IVCM examination of the same vessel reveals new sprouting from the hyper-dilated vessel starting on day 3 (arrows) developing into a perfused sprout with flow (arrows) on day 4. **e** A hyper-dilated vessel containing multiple sprouts (asterisks) along its length. **f** Slit lamp image on day 4 indicating new sprouts containing blood (arrows in magnified region of the cornea indicated by dashed white circle). **g** and **h** Immunostaining of new sprouts on day 4. **g** New sprouts (asterisks) express CD31 and weakly express Coll IV, while ebms are in close proximity (arrows) and strongly express Coll IV but not CD31. **h** An area with multiple new sprouts (asterisks) exhibiting strong CD31 expression but only weakly expressing Coll IV (arrows). Scale bars: **d** and **e** 50 µm, **g** 20 µm, **h** 100 µm

Dilation of the persistent vessels, evident from longitudinal slit lamp images of the same cornea (Fig. 4a–c), was followed by de novo sprouting from these vessels starting at 3 days after stimulation of revascularization, as observed by IVCM (Fig. 4d, e, Supplementary Video 2) and by slit lamp examination (Fig. 4f). By immunohistochemistry, new angiogenic sprouts emerging from the dilated persistent vessels expressed CD31 but only weakly expressed Coll IV by day 4, while ebms near and in contact with vessels did not appear to interact with new sprouts, maintaining strong expression of CoIl IV but not CD31 (Fig. 4g, h). In contrast to the revascularization phase where abandoned sprouts (ebms) expressed only Coll IV, immunostaining at day 7 of initial angiogenesis indicated that initially sprouts expressed both CD31 and Coll IV (Supplementary Fig. 1).

### Acellularity of ebms is maintained during revascularization

**Fig. 5:**
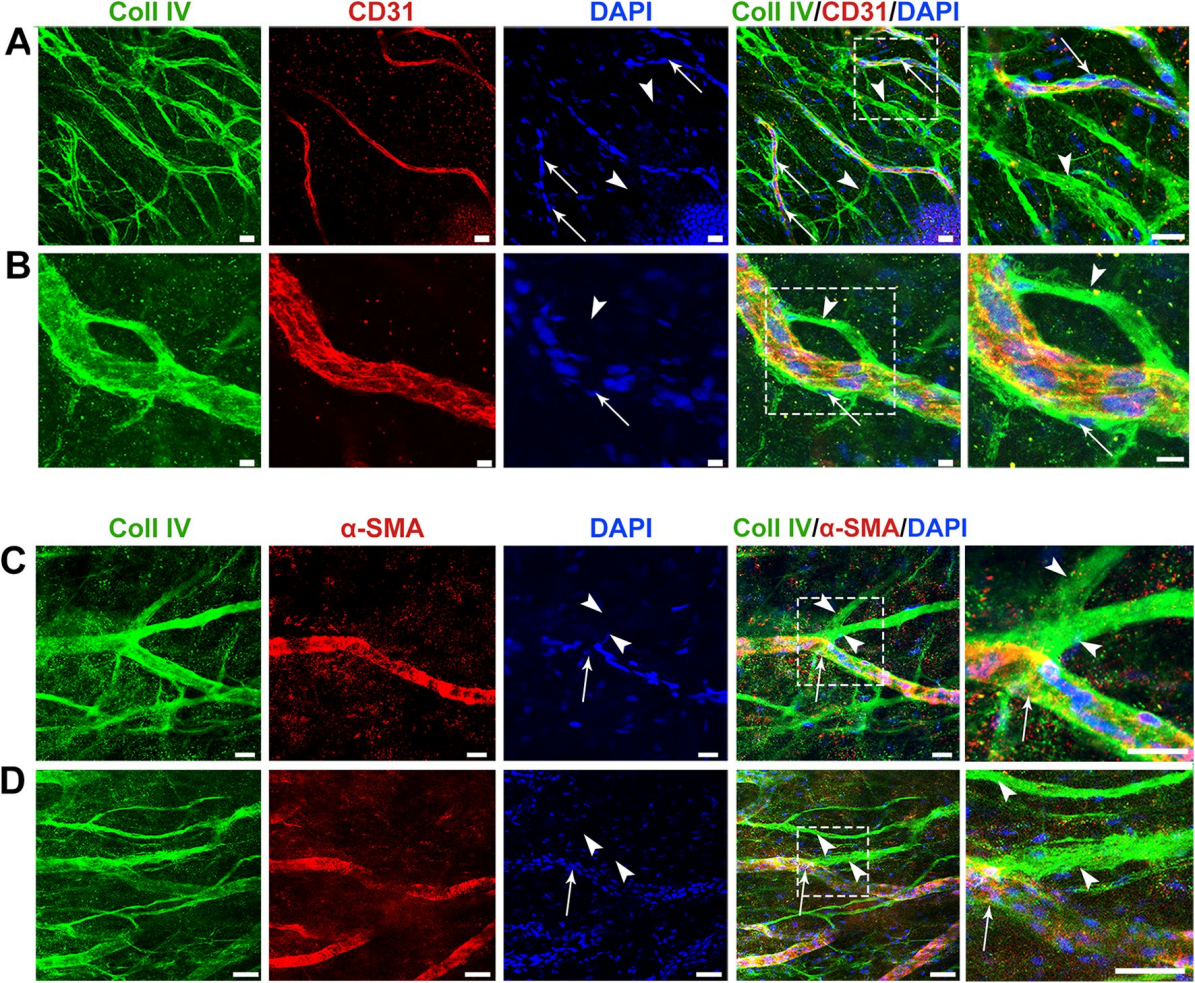
Acellularity of ebms is maintained during revascularization. **a** A dense network of ebms in the tissue consisted of many ebms (arrowheads) without co-localization of DAPI-stained cell nuclei. Persistent vessels additionally expressing CD31, however (arrows), had many associated cells along their length. **b** Persistent vessels had cells within or around the vessel wall (arrows) and a cobblestone structure of Coll IV and CD31 expression, while ebms (arrowhead) lacked this structure. **c** and **d** Ebms (arrowheads) did not co-localize with α-SMA expression, which was abundant in cell-covered persistent vessels. To the right of the merged images of **a**–**d** are magnified views of dashed white boxes in **a**–**d**, respectively, indicating cell-covered persistent vessels (arrows) and acellular ebms (arrowheads). Scale bars: **a**–**c** 20 µm, **d** 50 µm

To confirm the absence of vascular endothelium on ebms and to rule out the presence of any other cell type present on or within the ebms, Coll lV/CD31 and Coll lV/α-SMA staining were performed with DAPI counterstaining at 4 days of revascularization. Persistent vessels had a cellular structure indicated by a cobblestone morphology of CD31, Coll IV, and α-SMA staining, with abundant DAPI-stained cell nuclei attached to or within the vessel wall, along their entire length (Fig. 5a, b). α-SMA (Fig. 5c, d) expression of all cellular vessel structures further indicated pericyte coverage of these vessels (Fig. 5a–d). By contrast, ebms expressing Coll IV but lacking CD31 (Fig. 5a, b) and lacking α-SMA expression (Fig. 5c, d) also lacked a cobblestone morphology and notably did not have cell nuclei within or in contact with the sleeves (Fig. 5a–d). 3D image reconstruction and rendering of fluorescent confocal z-stack images confirmed the complete lack of cells on ebms, while adjacent persistent vessels expressing CD31 were cell-covered (Fig. 5, Supplementary Video 3).

### Excess collagen IV deposition irreversibly isolates ebms from parent vessels

**Fig. 6:**
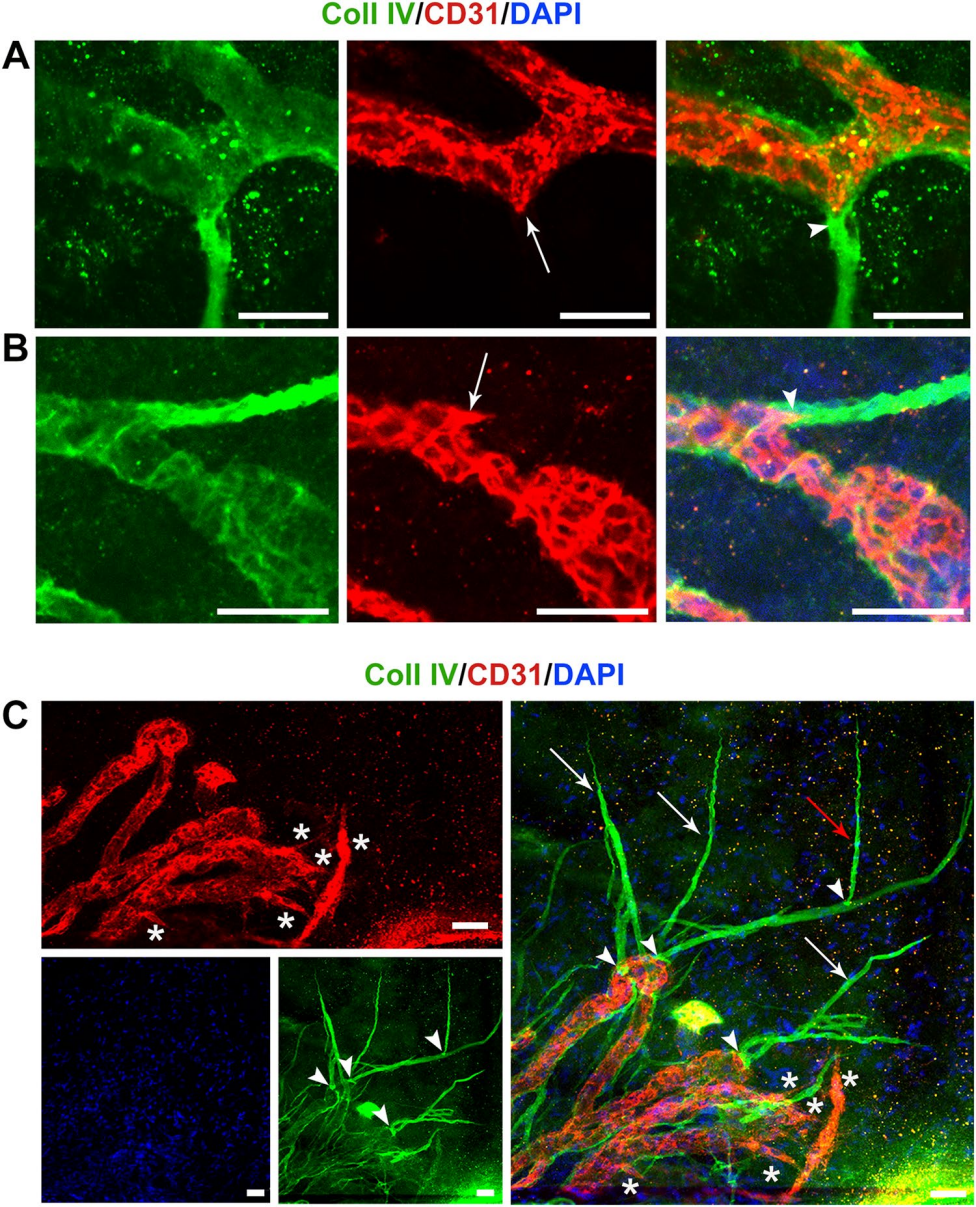
Differential distribution of basement membrane in new sprouting. **a**, **b** Localized, denser deposition of basement membrane within ebms (arrowheads) was observed proximally at the junction of persistent vessels and ebms. At locations where these basement membrane ‘plugs’ did not extend fully to the lumen of the persistent vessel, the EC cytoskeleton adapted to the form of the basement membrane (arrows) until the plug was encountered. **c** During the revascularization response, in a region of new sprouting, previously abandoned sprouts during the regression phase were transformed into ebms expressing Coll IV but not CD31 or DAPI (white arrows), with a previous secondary ebms visible (red arrow) extending from the primary ebms. Basement membrane plugs were observed at junction sites of primary and secondary ebms as well as at junction sites between persistent vessels and ebms (arrowheads). Vascular endothelium did not penetrate ebms beyond the plugs. De novo sprouting from persistent vessels (asterisks) occurred in close proximity to ebms, but unlike the strong Coll IV expression of ebms, the new sprouts had only very weak Coll IV expression consistent with delayed basement membrane deposition. Scale bars: **a** 20 µm, **b** and **c** 50 µm

Differential staining distribution of Coll IV was also noted. Basement membrane tended to be overexpressed particularly at junction points where the ebms of a regressed vessel was in contact with the parent persistent/revascularized vessel (Fig. 6). These regions of denser basement membrane deposition were interpreted as ‘plugs’ of compacted collagen appearing to block luminal access to the parent vessel (Fig. 6) and in some cases where plugs were apparent a short distance from the junction, the EC cytoskeleton adopted the shape of the junction a short distance into the ebms, until the dense collagen plug was encountered (Fig. 6a, b, Supplementary Video 4). The plugs thereby appeared to inhibit further EC repopulation of the ebms. In vigorous sprouting during initial angiogenesis, secondary sprouts emerge as extensions from primary sprouts; during rapid regression, both these primary and secondary sprouts transformed into ebms. At the junction points of primary and secondary sprouts, a basement membrane plug was also observed (Fig. 6c). During revascularization, de novo sprouting was morphologically distinct from ebms, with the new angiogenic sprouts emerging from persistent vessels and expressing CD31 (Fig. 6c). New sprouts had weak Coll IV staining indicative of their immature state, while ebms in close proximity had typical strong Coll IV expression and remained acellular (Fig. 6c).

### Persistent vessels are perfused, have EC and pericyte coverage, and EC rapidly normalize during revascularization

**Fig. 7:**
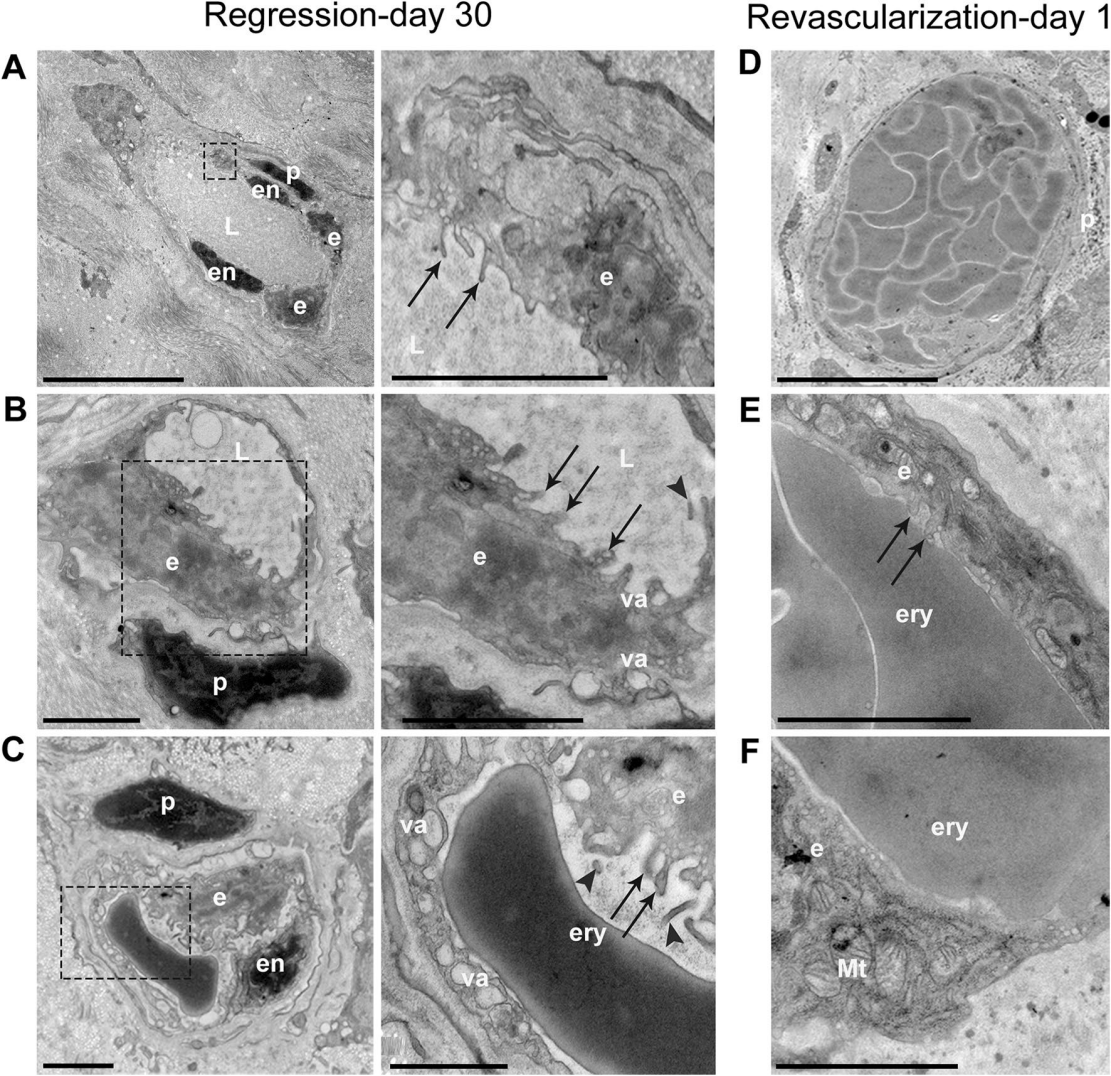
Ultrastructure of persistent vessels before and 1 day after revascularization stimulus. (**a**–**c**) Images of persistent vessels after 30 days of regression, with dashed boxes indicating the region of higher magnification shown in images to the right. **a** Plasma-filled vessel with retained pericyte (*p*) and EC (*e*) cytoplasmic processes (arrows) extending into the lumen (L). **b** Pericyte-covered persistent vessel with swollen EC (*e*) partially occluding the plasma-filled lumen (L), with cytoplasmic processes (arrows) and possible shedding of the EC into the lumen (arrowhead). Large cytoplasmic vacuoles are present (va). **c** Persistent vessel with pericyte (*p*) and swollen EC (*e*) partially occluding the lumen and permitting only serial erythrocyte (ery) flow. EC cytoplasmic processes (arrows) and possible EC shedding into the lumen (arrowheads) are visible. **d**–**f** Persistent vessels after 1 day of stimulation of revascularization. **d** Hyper-dilated pericyte-covered persistent vessel with many tightly packed erythrocytes filling the lumen. The pericyte (*p*) is in close contact to the vessel. **e**, **f** Magnified regions of a different persistent vessel, indicating smooth EC to luminal interface with few cytoplasmic processes (arrows) retracted into the cell body. Mitochondria (Mt) are visible in the cytoplasm. Scale bars: **a** 10 µm, 2 µm (zoomed), **b** 2 µm, 2 µm (zoomed), **c** 2 µm, 1 µm (zoomed), **d** 10 µm, **e** and **f** 2 µm. Labels: *ery* erythrocyte, *p* pericyte, *e* endothelial cell body, *en* endothelial cell nucleus, *L* lumen, *va* vacuole, *Mt* mitochondria

To further investigate structural transformations in persistent vessels in response to revascularization, the ultrastructure of these vessels before and after the revascularization stimulus was examined by transmission electron microcopy (TEM). After 30 days of regression (Fig. 7a–c), persistent vessels retained pericytes, EC, and a narrow lumen. These vessels had plasma flow punctuated by intermittent erythrocytes (Fig. 7a–c), confirming slit lamp observations (Figs. 2b, 3b). EC appeared in a degenerate form with swollen, bulging cell bodies containing large vacuoles and cytoplasmic processes extending into the lumen (as well as abluminal extensions), with some of these extensions appearing to be shed into the lumen (Fig. 7b, c). Partial EC occlusion of the lumen permitted only occasional single, serial erythrocyte flow (Fig. 7c). 1 day after the revascularization stimulus, persistent vessels increased in size and were re-perfused with many tightly packed erythrocytes (Fig. 7d–f). Re-perfused vessels were lined by slender EC with a smooth luminal interface and with very few cytoplasmic processes that were retracted into the cytoplasm (Fig. 7e, f). Pericytes appeared in looser association with vessels prior to revascularization but appeared to be in closer contact with the basement membrane during revascularization (Fig. 7a–d).

### A shorter course of angiogenesis inhibition results in incomplete vessel regression

**Fig. 8:**
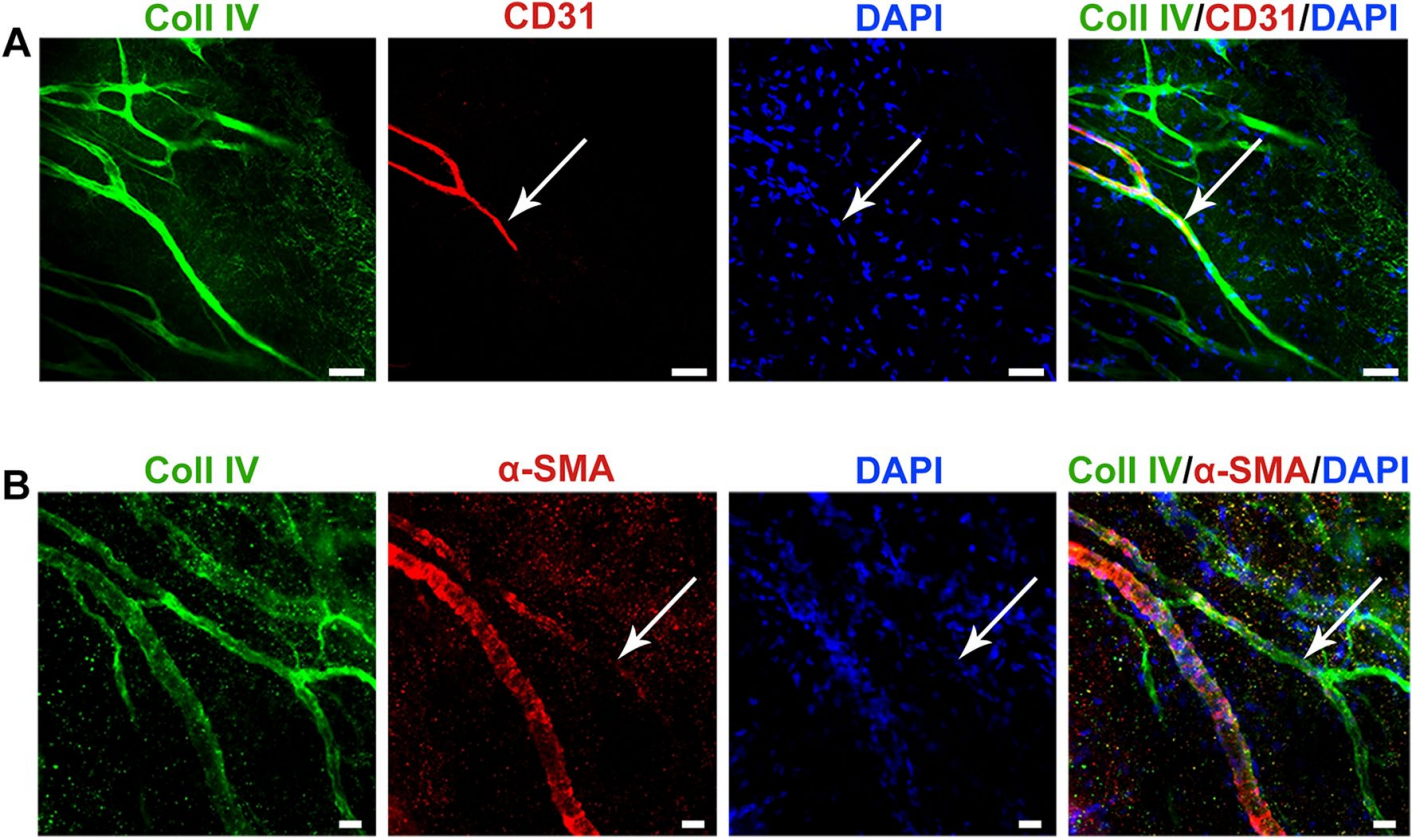
Partial regression after 7 days of angiogenesis inhibition **a** Immunostaining showing regressing vessels stained partially by CD31 (arrows), relative to the maintained Coll IV basement membrane. **b** Immunostaining indicating a regressing vessel (arrow) partially losing pericyte coverage (α-SMA expression) from the Coll IV basement membrane. Scale bar in A and B = 50 and 20 µm, respectively

To determine the effect of a shorter course of angiogenesis inhibition on vessel and ebms phenotype during revascularization, initial angiogenesis was again allowed to proceed over a 7-day period; however, on the seventh day, both sutures were removed to induce regression for only a 7-day period, instead of 30 days. After this short 7-day course of angiogenesis inhibition, immunostaining of the corneas revealed an expected incomplete regression of vessels, with Coll IV-expressing sleeves partially occupied by cells expressing CD31 and α-SMA (Fig. 8). Additionally, the sleeves were connected to the parent persistent vessels and to the circulation, without evidence of deposition of thick basement membrane at the connection point to the parent vessel.

## Discussion

A 30-day course of inhibiting angiogenesis achieved effective regression to dramatically regress new angiogenic vessels, restoring murine corneal transparency; however, a sub-population of barely visible vessels with markedly narrowed diameter and restricted flow persisted in the tissue. These persistent vessels facilitated revascularization of the tissue through a process of hyper-dilation and subsequent de novo sprouting. The persistent vessels remained in the tissue despite a sustained anti-angiogenic milieu, retaining pericyte coverage and an intact, although abnormal, vascular endothelium. Persistent vessels remaining in the tissue are a well-known phenomenon [51], and consistent with the hypothesis of vascular normalization of initial angiogenic vessels following anti-angiogenic treatment [52–54]. Despite this ‘normalization,’ however, we observed abnormalities at the ultrastructural level. Pericytes appeared swollen and partly detached from the vascular wall, while EC bulged and extended into the vessel lumen and formed luminal and abluminal processes. Flow was present in the persistent vessels but consisted of plasma/plasma-like substance interrupted by occasional erythrocytes flowing in a serial manner. These findings are consistent with earlier studies of vessel regression in other tissues [55–58], indicating a degeneracy of incompletely regressed vessels at the cellular level. Remarkably, however, this degeneracy reversed after discontinuation of the angiogenesis inhibition phase, with pericytes and EC of persistent vessels rapidly (within 24 h) reverting to a normal phenotype to permit increased perfusion and flow. This ‘reverse normalization’—in response to a sudden increase in vasodilating and angiogenic factors—re-activated the degenerate persistent vessels, to mount a strong revascularization response.

Interestingly, the early vasodilation effect we report in the current model has also been observed in other tissues. For instance, following 7 days of VEGF blockade in healthy mice, discontinuation of treatment induced dilation and bulging of hepatic vessels [9]. Likewise in tumor-bearing mice, 7-day VEGF blockade led to treatment-resistant vessels (persistent vessels) appearing dilated 2 days following treatment withdrawal [32]. Finally, in a patient receiving aflibercept injections for nAMD, longitudinal optical coherence tomography angiography imaging suggested that dilated persistent vessels comprised the revascularization response and led to continued pathology necessitating re-treatment [12].

Regression of vessels in the present model was associated with formation of a network of extensive ebms in the tissue as a record of the initial angiogenesis. Despite the abundance of ebms in direct contact with persistent vessels, there was no evidence to support their reuse by EC after a 30-day course of angiogenesis inhibition. While injury models of normally vascularized tissue such as the retina and skeletal muscle provide some evidence of partial ebms reuse [26–29], here we note that pathologic angiogenic vessels once fully regressed appear to remain irreversibly dormant. With reactivation of vasodilating/permeability factors such as VEGF-A, it may be more favorable for EC within the persistent vessels to reactivate to a functional state by normalizing and reconfiguring to accommodate an expanding lumen, rather than repopulate the acellular ebms by proliferation and migration. By contrast, if ebms were reused as conduits for EC, extremely fast EC proliferation and migration into ebms would have been required to account for the observed speed and extent of revascularization. In addition, at least a few partially colonized ebms would be present in the tissue during revascularization. None of these effects were observed during revascularization after a 30-day course of angiogenesis inhibition. In an earlier study of tumor revascularization after a short 7-day course of VEGF inhibition [32], α-SMA staining on ebms indicated that the structures may not have been acellular after a short duration of treatment. This effect was also observed in our model after only 7 days of angiogenesis inhibition; partially regressed vessels still had some EC and smooth muscle cell coverage and retained connection to the circulation. A very short course of anti-VEGF treatment may therefore lead to an intermediate stage of regression prior to full cellular abandonment of the vascular basement membrane, which could possibly support re-functionalization of partially regressed vessels upon abrupt discontinuation of the treatment.

By contrast, after a longer course of angiogenesis inhibition mimicking clinical time frames of anti-angiogenesis treatment, more vascular basement membrane had complete cellular abandonment leading to formation of ebms, with roughly equal distribution of ebms and persistent vessels in the tissue. Ebms, once formed, were unfavorable for repopulation by EC or pericytes during tissue revascularization. Based on our observations we suggest that during vascular regression, ebms are rendered irreversibly dormant by a pruning process of luminal closure at proximal sites by excess basement membrane deposition in the form of ‘plugs.’ These plugs exhibit strong type IV collagen expression, localize to the junction of ebms with persistent vessels, and could be deposited to prevent leakage from persistent vessels. Similar dense Coll IV plugs at junction points with perfused persistent vessels were apparent in a RIP-Tag2 tumor model [32], with the EC cytoskeleton closely following the form of the plugs but appearing not to penetrate them, as we also observed in the present model. In the tumor model, however, the intense Coll IV expression (plugs) was interpreted as “matrix heterogeneity accompanying new sprouts” [32]. Conversely in the present study, basement membrane in new sprouts (1–2 days old) had very weak to absent collagen IV expression, consistent with endothelial tip cell formation requiring basement membrane degradation [59]. Furthermore, earlier studies show Coll IV absence on new sprouts and deposition only in a later phase [60–62], consistent with new sprouts expressing Coll IV only at a later time point (7 days of sprouting) in our model. Observations of new Coll IV-negative angiogenic sprouts adjacent to plugged, dormant, and acellular Coll IV-positive ebms indicated that sprouting preferentially occurred away from the Coll IV plugs. EC cytoskeletal adaptation to the shape of the plugs moreover suggests Coll IV in ebms inhibiting EC migration, in accordance with known anti-angiogenic properties of Coll IV [63, 64].

By electron microscopy and α-SMA expression in this study, and using the pericyte marker NG-2 in prior studies [48, 65], pericyte coverage of persistent vessels was apparent, whereas ebms remained acellular during revascularization. This selectivity of vessel regression associated with pericyte coverage has similarly been reported in tumors [66].

It deserves mention that the model used to induce angiogenesis and regression in this study differs from the clinical situation of angiogenesis mediated by disease-related processes, followed by treatment with pharmacological inhibitors of angiogenesis. It would therefore be useful to verify the mode of revascularization in models more closely paralleling disease, and additionally using long-term pharmacologic inhibition of angiogenesis. Nevertheless, much knowledge pertaining to angiogenesis has been obtained through use of models selected to simplify and separate the complex processes occurring during the course of disease and its treatment.

**Fig. 9:**
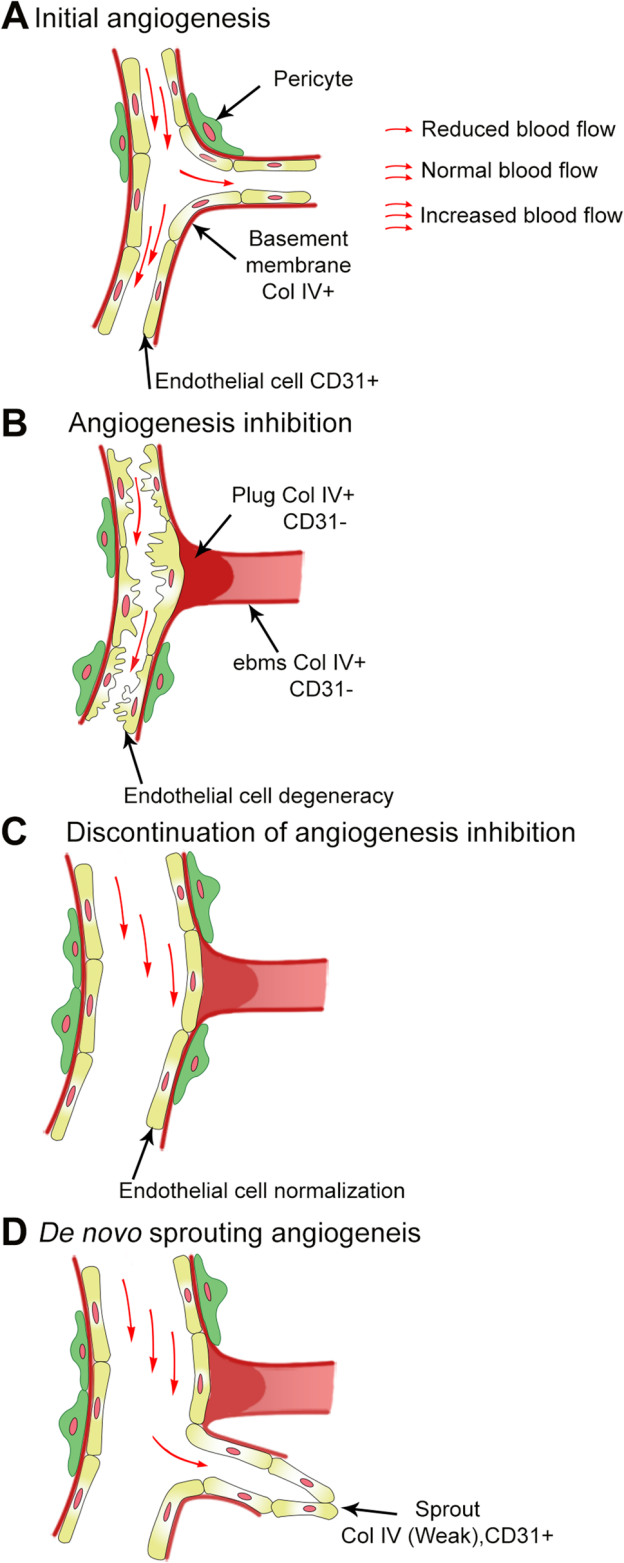
Schematic of the sequence of events characterizing revascularization observed in this study. **a** 1 week after initiation of angiogenesis (suture placement in the model), newly sprouted vessels are lined with EC expressing CD31, are perfused, and have a basement membrane expressing Coll IV and some acquire pericytes. **b** With prolonged anti-angiogenic treatment (suture removal in the model), blood flow in some vessels persists but is markedly reduced. EC in these persistent vessels swell and extend their cytoplasm into the lumen, further restricting the flow. Pericytes are retained on the persistent vessels. Other vessels regress by losing cellular coverage and CD31 expression, consisting only of an ebms expressing Coll IV. As the ebms loses cellular coverage at the point of connection to the blood flow, a dense plug of basement membrane is formed, isolating the ebms from the flow. **c** Upon removal of anti-angiogenic treatment (re-suturing in the model), EC within persistent vessels rapidly retract cytoplasmic extensions and regain a long, slender cytoskeleton with smooth luminal interface as the vessel hyper-dilates. Pericytes remain and flow increases significantly. The attached ebms remains dormant and acellular. **d** Several days following removal of anti-angiogenic treatment, new sprouting occurs from the hyper-dilated persistent vessel at sites away from the ebms, which remains acellular, dormant, and plugged. New sprouts initially consist of EC expressing CD31 but only weakly express Coll IV

In summary, we present an experimental model enabling longitudinal in vivo investigation of revascularization after inhibition of pathologic angiogenesis. Our findings indicate a sequence of events during regression and revascularization that are summarized schematically in Fig. 9. In our model, revascularization occurs principally through vasodilation and de novo sprouting from persistent vessels, with sprouting preferentially occurring away from fully abandoned ebms. Persistent vessels retain pericytes and EC and are primed for a rapid revascularization by re-normalizing in response to an angiogenic stimulus. Ebms, on the other hand, are remnants of regressed vessels that pervade the tissue, and once abandoned, they remain irreversibly isolated from flow by excess basement membrane deposition. These findings suggest EC recovery in persistent vessels, and not the dormant ebms, as a potential target to inhibit revascularization following cessation of angiogenesis inhibition.

## Methods

### Animal ethics and study model

Experiments were conducted after receiving approval from the Linköping Regional Ethical Committee for Animal Experiments, under ethical permit number 585. Experiments were also conducted with adherence to the guidelines of the Association for Research in Vision and Ophthalmology (ARVO) for the use of animals in ophthalmic and vision research. Wild-type male Wistar rats aged 5–6 weeks (Janvier Labs, France) were used for all experiments. The suture model of inflammatory corneal angiogenesis was used as previously described [47]. Briefly, two 10-0 nylon sutures were placed into the right eye cornea at 1.5 mm from the limbus. Sprouting of new blood vessels from the limbus toward central cornea occurred over a 7 days period (referred to as initial angiogenesis phase). On the 7th day, both sutures were removed from the cornea to induce vessel regression over a 30-day period (regression phase mimicking treatment). On the 30th day, the same cornea was again re-sutured with two nylon sutures placed at the same distance from the limbus as originally, to induce revascularization (revascularization phase), which was monitored longitudinally during a 4-day period, i.e., on days 0, 1, 3, and 4. In a separate experiment, the above procedure was repeated, however, terminating the experiment after only 7 days of regression.

### In vivo confocal microscopy (IVCM)

In vivo confocal microscopy (IVCM) (Heidelbert Retinal Tomograph 3 with Rostock Corneal Module HRT3-RCM, Heidelberg Engineering, Germany) was used as previously described [47], to monitor vessel perfusion and new sprouting. Serial images showing the same vessel at different time points were selected.

### Slit lamp imaging

To clinically monitor the overall neovascularization response, live images and video were captured using a rodent slit lamp [67] (Micron III, Phoenix Research Laboratories, USA). Vessels from still images were manually counted and compared between initial angiogenesis, regression, and revascularization phases.

### Whole-mount immunofluorescence staining

Rats were anaesthetized with a combination of ketamine (Pfizer) and dexdomitor (Orion Pharma) and euthanized by intracardial injection of pentobarbital. The cornea was dissected for whole-mount immunofluorescent staining. Briefly, the harvested cornea was embedded in OCT media (Thermo scientific) and frozen at -80 °C until use. Frozen samples were thawed at room temperature, washed in PBS for 1 h, and then fixed in cold acetone (− 20 °C) for 30 min. The fixed samples were washed three times in PBS for 30 min each time and blocked for 2 h with 10% normal goat serum at room temperature. Primary antibodies CD31 (1:300, MAB13932Z-Merck Milipore) and α-SMA (1:100, ab7817-Abcam) were added and incubated overnight at 4 °C. Subsequently samples were washed three times in PBS for 30 min each, and incubated with secondary antibody (1:100, DLlight 549-Abcam) overnight at 4 °C. For double staining with collagen IV, samples were then washed three times in PBS for 30 min each time and blocked for 2 h with 10% normal goat serum at RT, and then incubated overnight at 4 °C with primary antibody against Coll IV (1:300, ab19808-Abcam). After overnight incubation, samples were washed three times in PBS for 30 min each and incubated with secondary antibody (1:100, Alexa Flour 488-Abcam) overnight at 4 °C. Samples were then washed in PBS for 1 h and mounted using quick hardening antifade mounting media (Sigma) and imaged using a laser scanning fluorescent confocal microscope (LSM 700, Zeiss).

### Image processing and analysis

Fluorescence images were analyzed using Huygens software (Scientific volume imaging). Image files were deconvoluted in express mode, and using the generated images, co-localization analysis was performed using Costes threshold and Pearson’s correlation coefficient (r). IMARIS software (Bitplane) was used for 3D surface rendering of z-stack fluorescence confocal image files (LSM 700, Zeiss).

### Transmission electron microscopy

Harvested cornea samples were fixed in 2% glutaraldehyde in 0.1 M Na cacodylate, pH 7.4. Fixed samples were washed in the same buffer and post fixed in 2% Osmium tetroxide. Following en block staining with 2% uranyl acetate in 50% ethanol, samples were dehydrated in a series of ascending concentrations of ethanol and acetone. A three-step infiltration was performed prior to embedding in epoxy embedding medium kit (SIGMA-ALDRICH GmbH). Blocks were initially trimmed and sectioned using a Leica Ultracut UCT microtome (Leica UC7 ultra microtome (Leica Microsystems GmbH, Vienna, Austria). Ultrathin sections (70-nm thickness) were collected onto a formvar-coated copper slot grid, and counterstained with uranyl acetate and lead citrate. Images were taken using a 100 kV transmission electron microscope (EM JEM 1230, JEOL Ltd, Tokyo, Japan).

### Statistical analysis

The Shapiro–Wilk normality test was used with alpha = 0.05 to test for normal distribution. When comparing two sample means, the student t test was used for normally distributed data, while the non-parametric *Mann*–*Whitney U test* was used where data were not normally distributed. When comparing multiple groups, one-way ANOVA with Turkey’s post hoc multiple comparisons test was performed. A *P* value < 0.05 was considered significant. The Pearson’s correlation coefficient and the Costes threshold were used in the co-localization analysis.

## Electronic supplementary material

Below is the link to the electronic supplementary material.

Supplementary material 1 (DOCX 6427 kb)

Supplementary material 2 (MP4 2033 kb)

Supplementary material 3 (MP4 2406 kb)

Supplementary material 4 (MOV 11030 kb)

Supplementary material 5 (MPG 1760 kb)

Supplementary material 6 (TIFF 4740 kb)

Supplementary material 7 (TIFF 6341 kb)
