# Supplementary material for: Revascularization after angiogenesis inhibition favors new sprouting over abandoned vessel reuse
Source: Angiogenesis. 2019 Sep 4;22(4):553–67. doi: 10.1007/s10456-019-09679-9 (PMC6863948; doi:10.1007/s10456-019-09679-9)
Supplement: Supplementary file 1 — Supplementary material 1 (DOCX 6427 kb) [file 10456_2019_9679_MOESM1_ESM.docx]

**Supplementary material**

**Revascularization after angiogenesis inhibition favors new sprouting over abandoned vessel reuse**

Anthony Mukwaya^1^, Pierfrancesco Mirabelli^1^, Anton Lennikov^1,2^, Muthukumar Thangavelu^1,3^, Maria Ntzouni^4^, Lasse Jensen^5^, Beatrice Peebo^1^, and Neil Lagali^1,6^*

^1^Department of Ophthalmology, Institute for Clinical and Experimental Medicine,

Faculty of Health Sciences, Linkoping University, Linköping, Sweden

^2^Mason Eye Institute, Ophthalmology-Retinal Vascular Service Hospital MA102C, Missouri, MO USA.

^3^Dept BIN Convergence Tech & Dept PolymerNano Sci & Tech, Chonbuk National University, Jeonju, Republic of Korea

^4^Electron Microscopy and Histology Laboratory, Faculty of Medicine, Linköping University, Linköping, Sweden

^5^Department of Medical and Health Sciences, Division of Cardiovascular Medicine, Linköping University, Linköping, Sweden

^6^Department of Ophthalmology, Sørlandet Hospital Arendal, Arendal, Norway


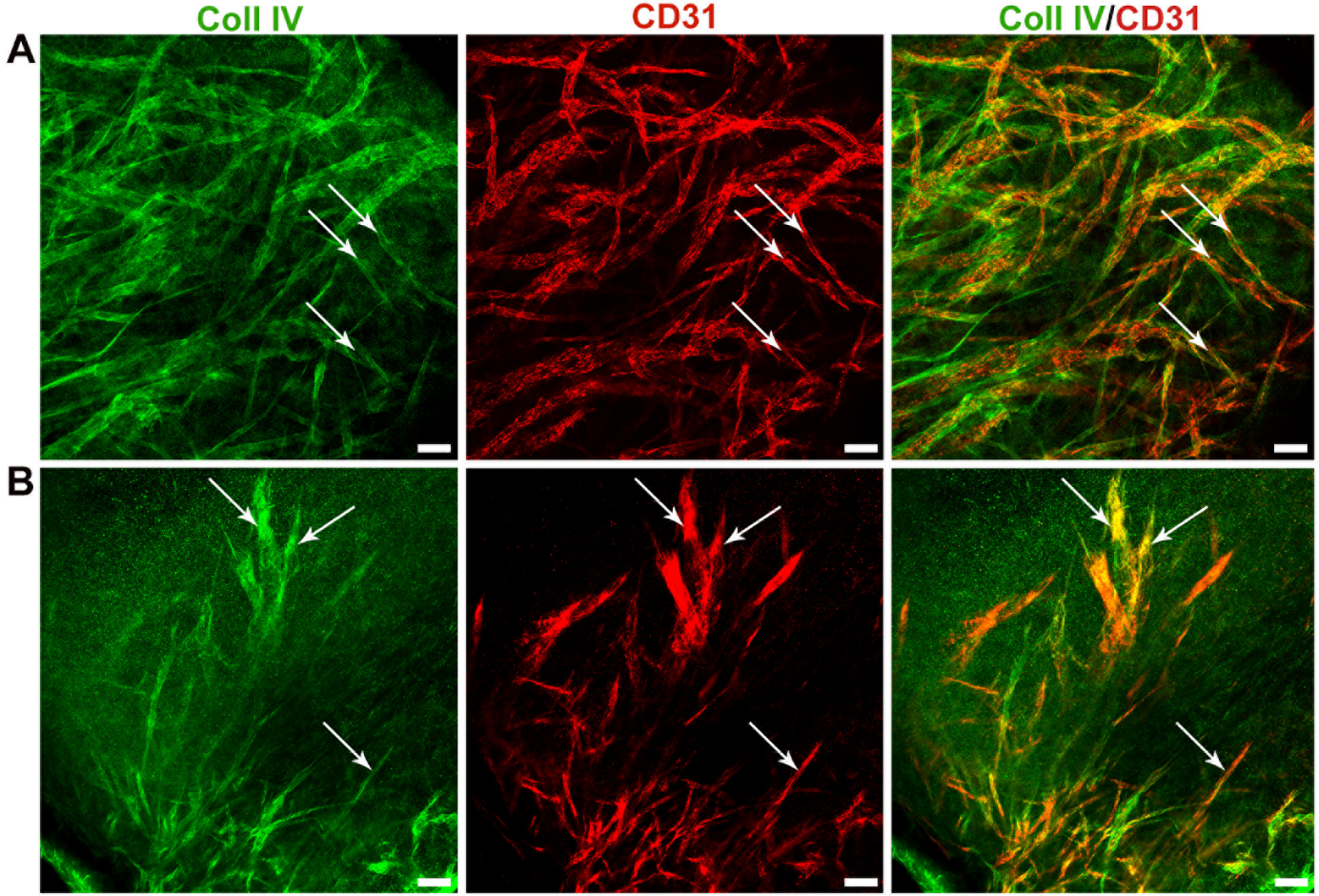


**Supplementary Figure 1. Immunofluorescent staining of cornea on day 7 of initial angiogenesis.** (**A** and **B**) Two regions at the front of vessel invasion during initial sprouting angiogenesis indicate many new vessel sprouts present (arrows), co-staining for vascular endothelium (CD31) and basement membrane (Coll IV). The same vessel sprouts are indicated in the different channels by arrows. Scale bars: 50µm.


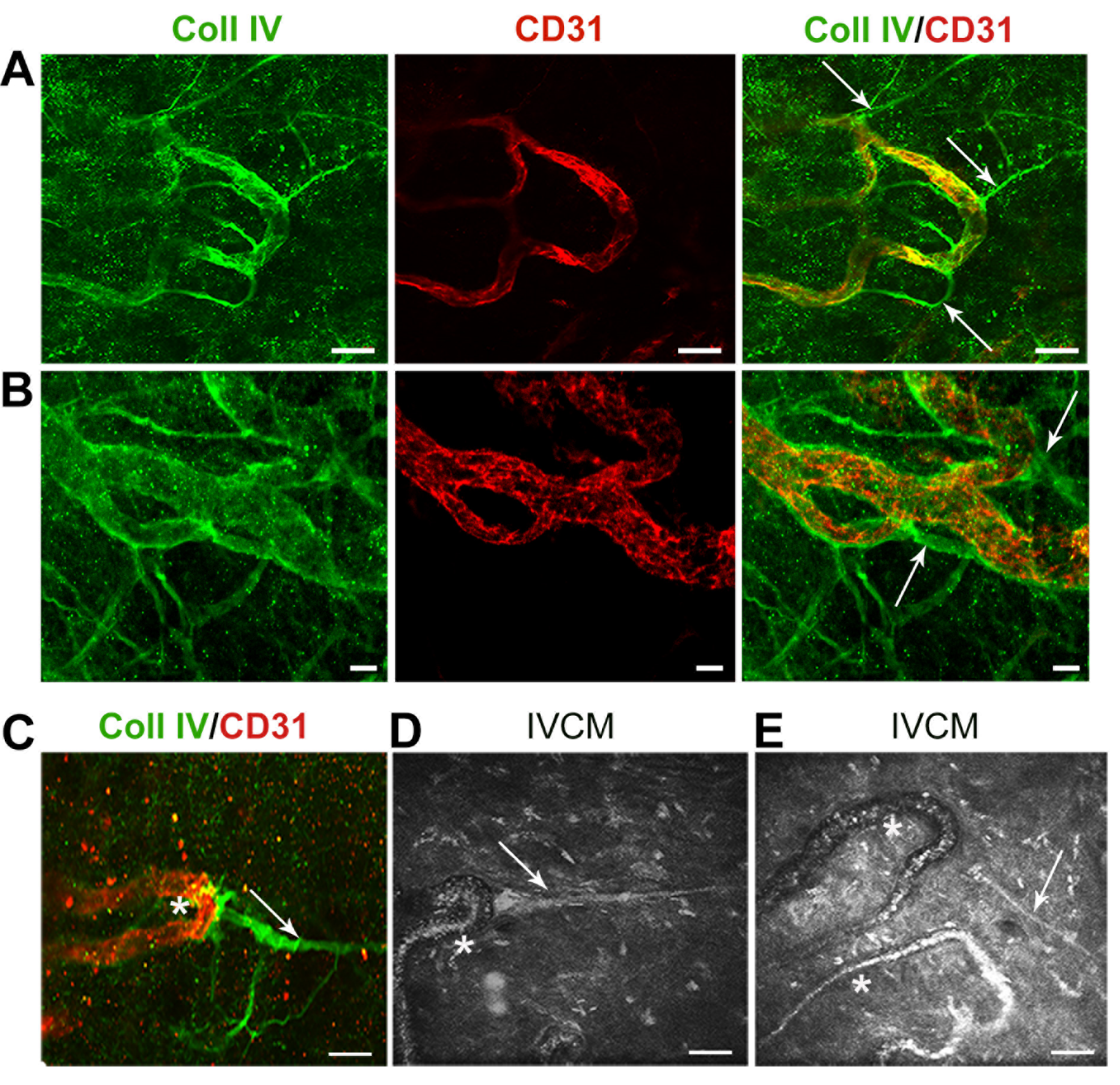


**Supplementary Figure 2. Absence of vascular endothelium on ebms.** (**A**) Immunostaining of vascular loops at the revascularization front shows only vascular loops are covered by endothelium while ebms were visible as thin strand-like connecting segments (arrows) lacking CD31 expression. (**B**) In the central revascularized region ebms were similarly observed as conduits (arrows) lacking CD31 expression. (**C**) a persistent vessel loop expressing Coll IV and CD31 (asterisk) with an associated ebms (arrow) (**D**) In the same tissue region observed with IVCM, the loop is perfused (asterisk) while the ebms (arrow) appears dormant. (**E**) In another cornea, IVCM similarly shows perfursed loops (asterisks) and associated dormant ebms (arrow). Scale bars: (A and C) 20µm, (B) 10µm (D and E) 50µm.


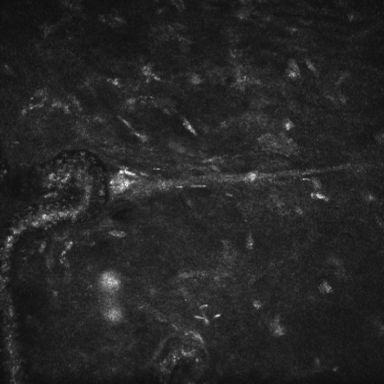


**Supplementary Video 1. Live imaging of non-perfused ebms during revascularization.** IVCM examination of a live rat cornea at day 4 of revascularization, indicating increased flow in hyper-dilated persistent vessels and attached ebms (arrow) that remains non-perfused. Note that motion artifacts are present due to animal respiration.


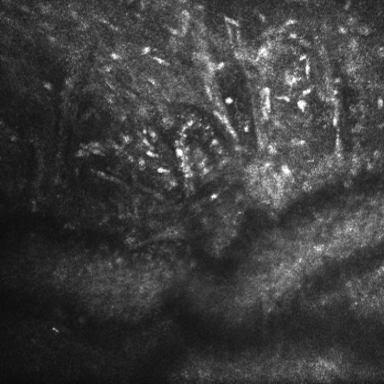


**Supplementary Video 2. Live imaging of de novo sprouts emerging from hyper-dilated persistent vessel during revascularization.** IVCM examination of a live rat cornea at day 4 of revascularization, indicating several new sprouts (arrows) emerging laterally from a hyper-dilated persistent vessel. Some flow and cells are visible within the sprouts. Note that motion artifacts are present due to animal respiration.


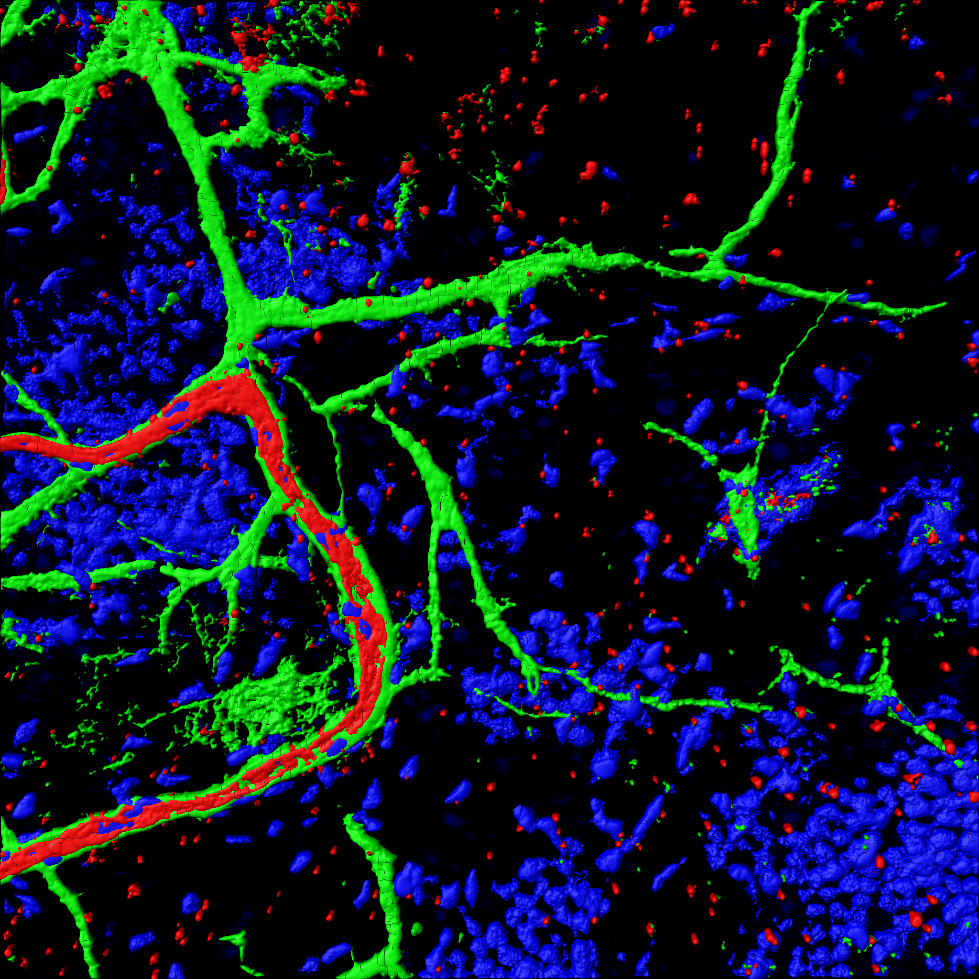


**Supplementary Video 3. 3D rendered confocal flourescence z-stack of persistent vessels and ebms after four days of revascularization.** The video shows the presence of DAPI-stained cell nuclei (blue) on CD31 expressing (red) persistent vessels and in the extravascular space, but an absence of cell nuclei on ebms which expressed only Coll IV (green).

**
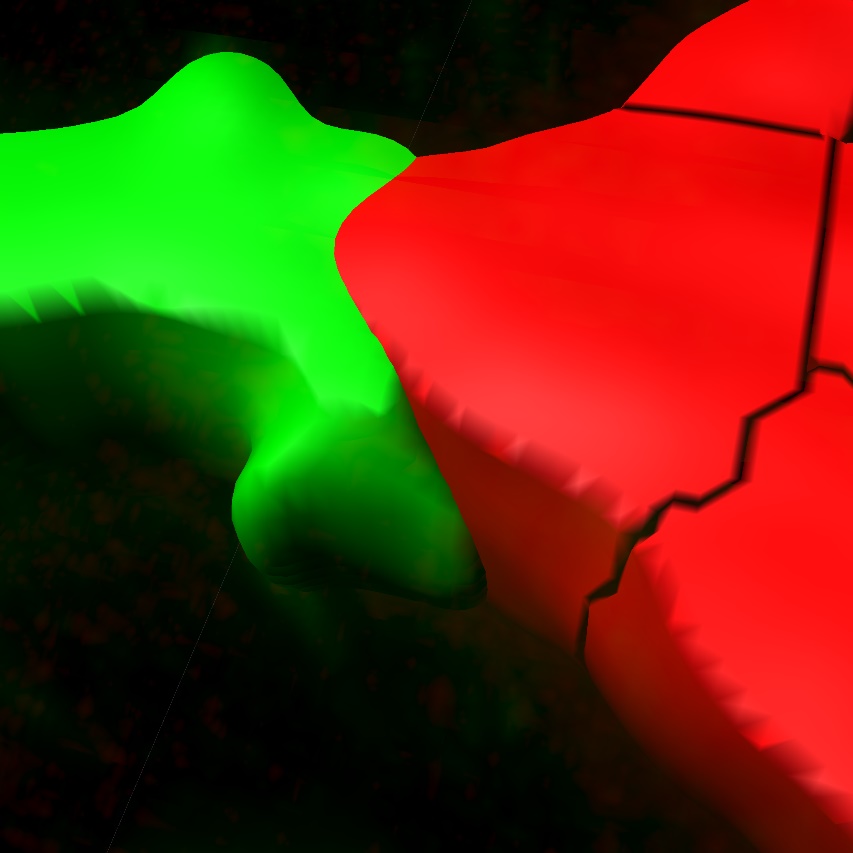
**

**Supplementary Video 4. 3D rendered confocal flourescence z-stack after four days of revascularization at an ebms junction with a persistent vessel.** The video shows the dense Coll IV (green) plug of the ebms appearing to hinder the migration of endothelial cells of persistent vessels expressing CD31 (red).
